# Diagnostic and prognostic nomograms for bone metastasis in hepatocellular carcinoma

**DOI:** 10.1186/s12885-020-06995-y

**Published:** 2020-06-01

**Authors:** Chuan Hu, Jiaxin Yang, Zhangheng Huang, Chuan Liu, Yijun Lin, Yuexin Tong, Zhiyi Fan, Bo Chen, Cailin Wang, Cheng-Liang Zhao

**Affiliations:** 1 Department of Orthopedic, Affiliated Hospital of Chengde Medical University, No. 36 Nanyingzi St., Chengde, 067000 Hebei China; 2 Qingdao University medical college, Qingdao, China; 3 Wenzhou Medical University, Wenzhou, China; 4 Graduate School of China Medical University, Liaoning, China; 5 School of Basic Medical Sciences, North China University of Science and Technology, Tangshan, China

**Keywords:** Hepatocellular carcinoma, Bone metastasis, Prognosis, Nomogram

## Abstract

**Background:**

Bone metastasis (BM) is one of the common sites of hepatocellular carcinoma (HCC), and the prognosis of BM patients is worse than patients without it. Our study aimed to identify predictors and prognostic factors of BM in HCC patients and develop two nomograms to quantify the risk of BM and the prognosis of HCC patients with BM.

**Methods:**

We retrospectively reviewed the data of patients who were diagnosed as HCC between 2010 and 2015 in the Surveillance, Epidemiology, and End Results (SEER) database. Independent predictors for BM from HCC patients were determined by the univariate and multivariate logistic regression analysis. Independent prognostic factors for HCC patients with BM were identified by univariate and multivariate Cox regression analysis. Two nomograms were established and evaluated by calibration curves, receiver operating characteristic (ROC) curve, and decision curve analysis (DCA).

**Results:**

Nine thousand and forty-seven patients were included. The independent risk factors of BM in newly diagnosed HCC patients are sex, grade, T stage, and N stage. The independent prognostic factors for HCC patients with BM are radiotherapy, chemotherapy, and lung metastasis. The AUC of diagnostic nomogram were 0.726 in the training set and 0.629 in the testing set. For the prognostic nomogram, the AUCs of 6-, 9-, and 12-months were 0.753, 0.799, and 0.732 in the training set and 0.698, 0.770, and 0.823 in the validation set. The calibration curve and DCA indicated the good performance of the nomogram.

**Conclusions:**

Two nomograms were established to predict the incidence of BM in HCC patients and the prognosis of HCC patients with BM, respectively. Both nomograms have satisfactory accuracy, and clinical utility may benefit for clinical decision-making.

## Background

Hepatocellular carcinoma (HCC) is one of the most common primary malignant tumors and the fourth leading cause of cancer-related death worldwide, with 841,000 new cases and at least 780,000 deaths in 2018 [1]. Nowadays, surgical excision, liver transplantation, local administration of radiation or chemical drugs, and combined therapy are the main treatments for HCC patients [2]. However, most patients are diagnosed in the advanced stage. Therefore, the prognosis remains poor, and the 5-year survival rate was less than 20% [3]. Moreover, 14.0–36.7% of patients have distant metastasis at initial diagnosis [4, 5], and the prognosis was poorer in HCC patients with extrahepatic metastasis than patients without it [6].

Bone metastasis (BM) is a typical metastatic pattern in HCC patients. It was reported that the incidence of BM in HCC patients ranged from 3 to 20%, and showed a rising trend [3, 7–9]. Although the management of HCC patients has improved in recent years, the prognosis of HCC with BM is abysmal, with a median survival of only 1–2 months [7]. Therefore, it is important to establish predictive models for predicting the BM in HCC patients and the prognosis in HCC patients with BM. In the previous studies, many risk factors and prognostic variables were identified, including SREs, AFP, Tomita scoring system, BMI, marriage status, and surgical treatment history of primary liver lesions [10–14]. However, no researches focused on the predictive model for predicting the BM in HCC and the prognosis of HCC with BM, which means that the probability of outcome cannot be quantified.

Nomogram is a simple, multivariate visualization tool in oncology to predict and quantify the rate of the outcome of an individual patient [15], which were used to aid clinical decisions and promote the development of precision medicine. Therefore, based on the data from the Surveillance, Epidemiology, and End Results (SEER) database, we aimed to develop two nomograms for predicting the BM in newly diagnosed HCC patients and the cancer-specific survival (CSS) of HCC patients with BM, respectively.

## Methods

### Study population selection

The data included in the present study were downloaded from the SEER*Stat software version 8.3.6. The analysis of the unidentified data from the SEER database was exempted from medical ethics review and didn’t require informed consent. The inclusion criteria were following: (1) Patients were histologically diagnosed as HCC from 2010 to 2015; (2) Demographic variables, including age, race, and sex were available; (3) Tumor characteristics, including histological grade, T stage, N stage, bone metastasis status were available. In addition, patients diagnosed with autopsies or death certificates were excluded from the present study. Finally, 9047 patients were used to form a cohort to study the risk factors of BM in HCC patients and establish a predictive nomogram. Afterward, HCC patients with BM with survival time ≥ one month, specific metastasis data, including liver metastasis, lung metastasis, and brain metastasis, and specific treatment information, including surgery, radiotherapy, and chemotherapy, were used to form a new cohort to explore the prognostic factors for HCC patients with BM and develop a prognostic nomogram. Ultimately, 190 patients were used to study the prognostic factors of HCC with BM. For each cohort, patients were randomly divided into the training set(70%) and testing set(30%). In the present study, patients in the training set were used to develop the nomogram, and patients in the testing set were used to validate it.

### Data collection

In the present study, seven variables were used to identify the risk factors of BM from HCC, including age, sex, race, grade, T stage, and N stage. For the study about the prognostic factors for HCC patients with BM, three treatment variables, including surgery (Performed or not performed), chemotherapy (Performed or not performed), and radiotherapy (Performed or not performed), and metastasis data, including liver metastasis(Yes or no), lung metastasis, and brain metastasis, were also included. In this part, CSS was the primary outcome, which was defined as the time interval between the day of diagnosis and the day of death due to cancer.

### Statistical analysis

All statistical analysis in our present study was conducted with SPSS 25.0 and R software (version 3.6.1). The chi-square test was used to compare the variables between the training set and the testing set. In the present study, a *p*-value < 0.05(two sides) was considered as statistical significance. Univariate logistic analysis was applied to identify BM-related factors. The variables with *p* value< 0.05 in the univariate logistic analysis were included in the multivariate binary logistic regression analysis to determine independent risk factors of BM in initially diagnosed HCC patients. For prognostic factors, the univariate Cox regression analysis was applied to identify prognostic variables. Then, significant variables in the univariate Cox regression analysis were incorporated into the multivariate Cox regression analysis, and the independent prognostic factors of HCC with BM were identified.

The predictive and prognostic nomograms were developed by the “rms” package in R software based on the independent predictive factors and prognostic factors, respectively [16]. Meanwhile, the receiver operating characteristic (ROC) curve for the predictive nomogram and the time-dependent ROC curve for the prognostic nomogram were generated [17]. The area under the curve (AUC) was used to evaluate the discrimination of nomograms. In addition, ROC curves or time-dependent ROC curve of all independent variables were also generated, AUCs of all independent variables were compared with the AUC of the nomogram. Moreover, the calibration curves and decision curve analysis (DCA) curves were established for the nomogram [18]. Finally, according to the median of risk score, all patients were divided into the high-risk and low-risk groups, and the survival curve with a log-rank test was used to verify the prognostic value of nomogram [19].

## Results

### The characteristics of the study population

According to the selection process, a total of 9047 patients were included in our research. Meanwhile, 6335 patients were incorporated into the training set, and the remaining 2712 patients were incorporated into the testing set. The baselines of 9047 patients were shown in Table 1.

**Table 1:** Clinical and pathological features of patients diagnosed as HCC

	Training set(6335)	Validation set(2712)	χ^2^	P
Age			0.053	0.818
<65	3534 (55.8%)	1520 (56.0%)		
≥ 65	2801 (44.2)	1192 (44.0%)		
Sex			7.001	0.008
Male	4813 (76.0%)	2130 (78.5%)		
Female	1522 (24.0%)	582 (21.5%)		
Race			1.314	0.518
African-American	885(14.0%)	403(14.9%)		
White	4230(66.8%)	1799(66.3%)		
Other	1220(19.3%)	510(18.8%)		
Grade			0.177	0. 674
G1–2	4915(77.6%)	2115(71.3%)		
G3–4	1420(22.4%)	597(28.7%)		
T stage			1.312	0 .252
T1–2	4590 (72.5)	1933(71.9%)		
T3–4	1745(27.5%)	779 (28.1%)		
N stage			0.255	0.614
N0	5896(93.1)	2532 (93.4%)		
N1	439(6.9%)	180 (6.6%)		
Bone metastasis			2.303	0.129
No	6183(97.6%)	2632(97.1%)		
Yes	152(2.4%)	80(2.9%)		

### Risk factors of bone metastasis in HCC patients

In 9047 patients, 232 cases (2.6%) with BM at initial diagnosis and 8815 cases (97.4%) without it. To identify BM-related variables in HCC patients, univariate logistic analysis was used to analyze seven predictors. The results revealed that five predictors were related to BM in HCC patients, including race, sex, histological grade, T stage, and N stage (Table 2). Then, the multivariate logistic regression analysis showed that male patients (*P* = 0.023), higher grade (*P* = 0.002), higher T stage patients (*P* < 0.001), and higher N stage patients (P < 0.001) were independent predictors of BM in newly diagnosed HCC patients (Table 2).

**Table 2:** Logistic analysis of risk factor of BM in HCC patients

	Univariate analysis			Multivariate analysis		
	OR	95%CI	P	OR	95%CI	P
Age						
< 65						
≥ 65	0.941	0.680–1.303	0.715			
Race						
African-American						
Other	0.442	0.244–0.801	0.007			
White	0.751	0.495–1.141	0.180			
Sex						
Female						
Male	1.893	1.200–2.985	0.006	1.705	1.076–2.700	0.023
Grade						
I-II						
III-IV	2.379	1.711–3.309	0.000	1.719	1.220–2.424	0.002
T stage						
T1–2						
T3–4	3.653	2.636–5.062	0.000	2.607	1.841–3.691	0.000
N stage						
N0						
N1	5.177	3.558–.533	0.000	3.049	2.041–4.553	0.000

### Development and validation of a diagnostic nomogram for BM in newly diagnosed HCC patients

**Fig. 1:**
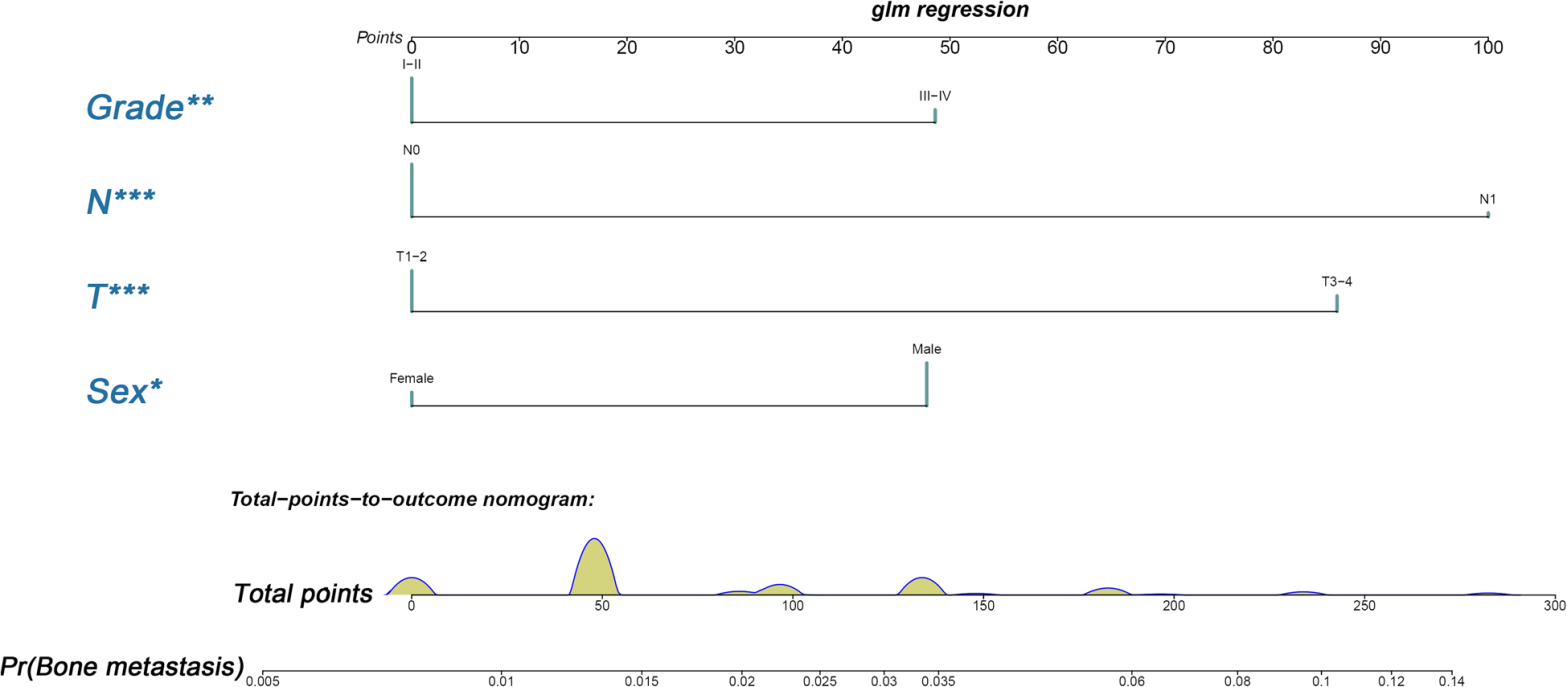
Nomogram for predicting BM from HCC patients

**Fig. 2:**
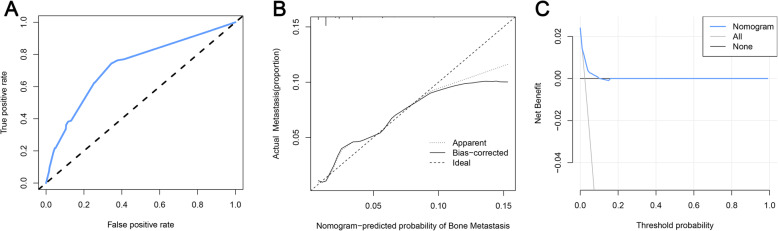
The receiver operating characteristic curve (**a**), calibration curve (**b**), and decision curve analysis (**c**) of the training set

**Fig. 3:**
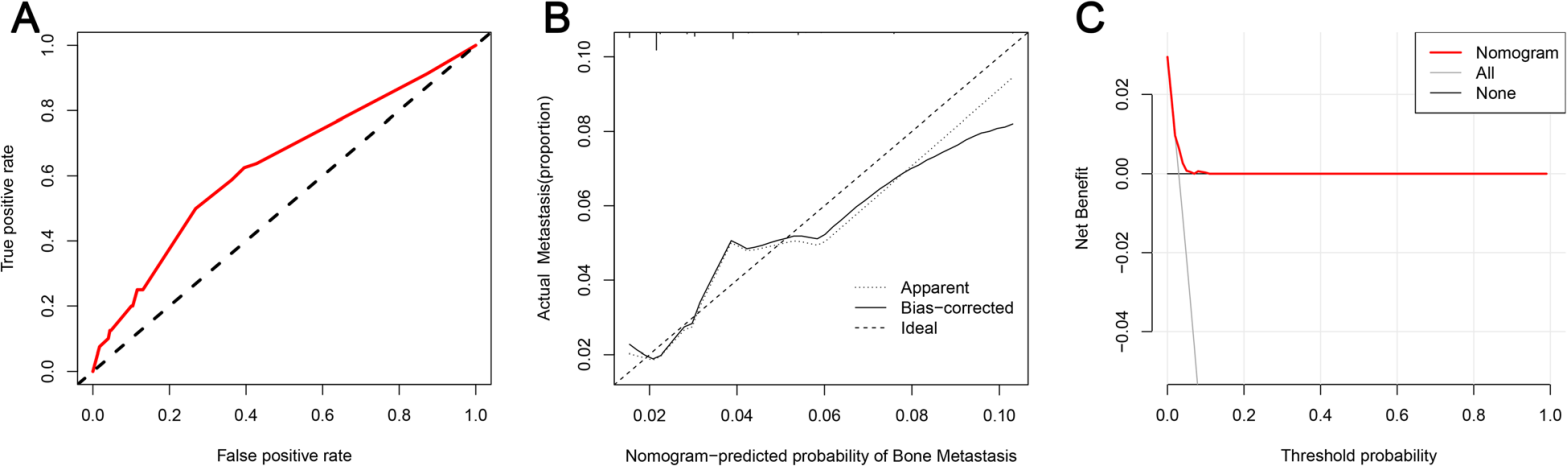
The receiver operating characteristic curve (**a**), calibration curve (**b**), and decision curve analysis (**c**) of the testing set

**Fig. 4:**
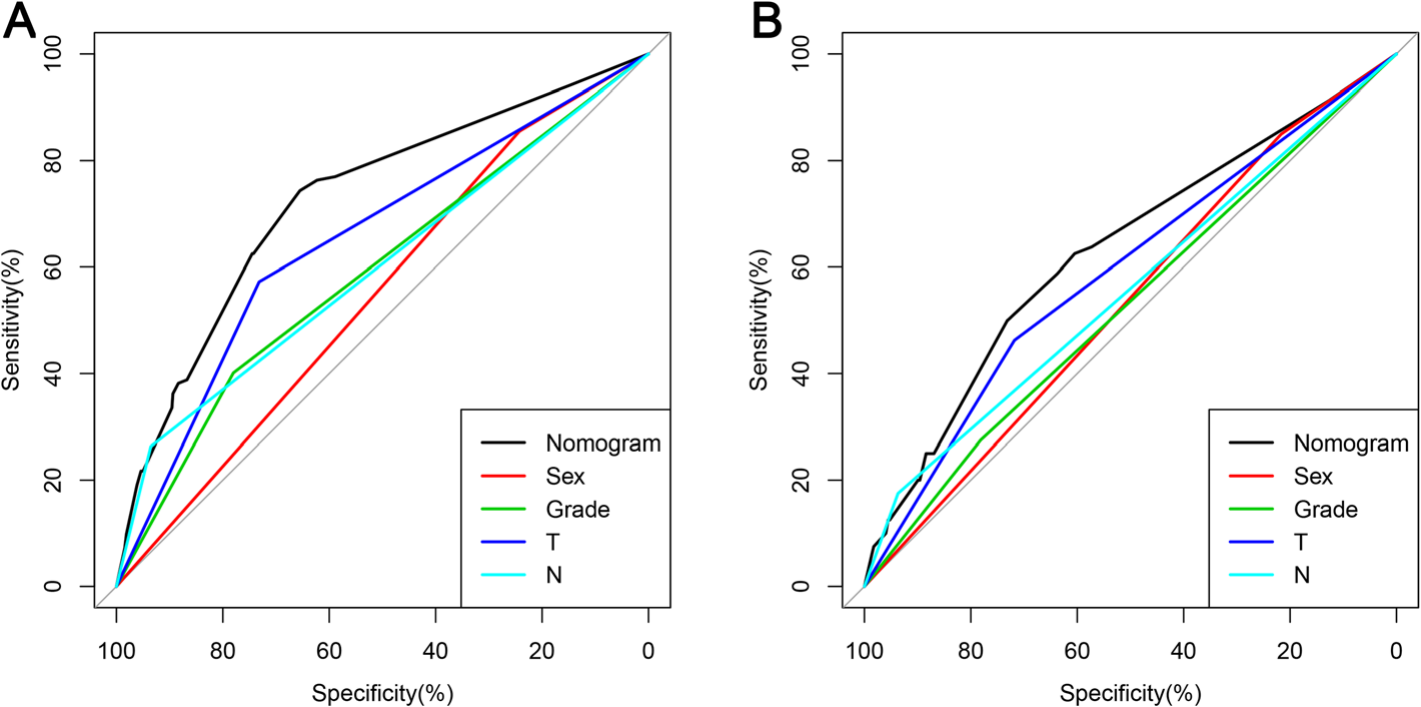
Comparison of area under the receiver operating characteristic curve between nomogram and each independent predictors in the training set (**a**) and the testing set (**b**)

Based on the four independent BM-related variables, a diagnostic nomogram was established for the risk assessment of BM in newly diagnosed HCC patients (Fig. 1). Meanwhile, the ROC curves of both training set and testing set were established, and the AUC of nomogram were 0.726 in the training set and 0.629 in the testing set (Figs. 2a and 3a). More importantly, the ROC curves of each independent predictors were also generated (Fig. 4). The results showed that the AUC of all predictors alone were lower than the AUC of nomogram, no matter in the training set and testing set. Furthermore, both in the training set and testing set, the calibration curves showed a robust calibration of nomogram (Figs. 2b and 3b), and DCA indicated that this nomogram can serve as an excellent diagnostic tool for BM in newly diagnosed HCC patients(Figs. 2c and 3c).

### Prognostic factors for HCC patients with BM

As shown in Table 3, 190 eligible HCC patients with BM were used to study the prognostic factors. Among 190 patients, the male patients (85.2%) were more than female patients (14.8%). And the race of 120 (63.2%) patients were White, 46 (24.2%) patients were Africa American, and 24 (12.6%) patients were other. Meanwhile, 133 patients were randomly divided into the training set, and the remaining 57 patients were incorporated into the testing set. The chi-square test showed that there were no significant differences between the training set and the testing set (Table 3).

**Table 3:** Clinical and pathological features of patients diagnosed as HCC with BM

	Training set	Validation set	X^2^	P
Age			0.017	0.898
<65	55(41.4%)	23(40.4%)		
≥ 65	78(58.6%)	34(59.6%)		
Sex			1.099	0.294
Male	114(85.7%)	52(91.2%)		
Female	19(14.3%)	5(8.8%)		
Race			0.502	0.778
Africa American	34(25.6%)	12(21.1%)		
Other	16(12.0%)	8(14.0%)		
White	83(62.4%)	37(64.9%)		
Grade			0.364	0.547
G1–2	90(67.7%)	36(63.2%)		
G3–4	43(32.3%)	21(36.8%)		
T stage			2.053	0.152
T1–2	64(48.1%)	21(36.8%)		
T3–4	69(51.9%)	36(63.2%)		
N stage			0.631	0.427
N0	105(78.9%)	42(73.7%)		
N1	28(21.1%)	15(26.3%)		
Surgery			1.007	0.316
No	126(94.7%)	51(89.5%)		
Yes	7(5.3%)	6(10.5%)		
Radiation			0.581	0.446
No	64(48.1%)	24(42.1%)		
Yes	69(51.9%)	33(57.9%)		
Chemotherapy			0.103	0.748
No	57(42.9%)	23(40.4%)		
Yes	76(57.1%)	34(59.6%)		
Brain metastasis			0.004	0.948
No	124(93.2%)	54(94.7%)		
Yes	9(6.8%)	3(5.3%)		
Liver metastasis			1.719	0.190
No	119(89.5%)	55(96.5%)		
Yes	14(10.5%)	2(3.5%)		
Lung metastasis			0.603	0.437
Yes	103(77.4%)	47(82.5%)		
Yes	30(22.6%)5	10(17.5%)		

As shown in Table 4, the univariate and multivariate Cox proportional hazard regression were performed to screen prognostic factors, which revealed that age, radiotherapy, chemotherapy, and lung metastasis were CSS-related factors, while the radiotherapy(*P* = 0.001), chemotherapy (*P* < 0.001), and lung metastasis (P < 0.001) were independently prognostic factors for HCC patients with BM.

**Table 4:** Univariate and multivariate Cox analysis in HCC patients with BM

	Univariate Cox analysis				Multivariate Cox analysis			
	HR	95%CI		P	HR	95%CI		P
Age								
< 65								
≥ 65	0.756	0.528	1.084	0.128				
Race								
African-American				0.840				
Other	1.134	0.594	2.164	0.704				
White	0.953	0.633	1.433	0.816				
Sex								
Female								
Male	0.789	0.463	1.342	0.381				
Grade								
I-II								
III-IV	1.403	0.960	2.050	0.080				
T stage								
T1–2								
T3–4	1.233	0.865	1.758	0.247				
N stage								
N0								
N1	1.242	0.797	1.934	0.339				
Surgery								
No								
Yes	0.699	0.306	1.594	0.394				
Radiation								
No								
Yes	0.535	0.371	0.771	0.001	0.597	0.41	0.871	0.007
Chemotherapy								
No								
Yes	0.555	0.386	0.799	0.002	0.623	0.428	0.906	0.013
Brain metastasis								
No								
Yes	0.847	0.412	1.742	0.652				
Liver metastasis								
No								
Yes	1.508	0.860	2.644	0.151				
Lung metastasis								
No								
Yes	1.623	1.071	2.458	0.022	1.528	1.003	2.328	0.048

### Prognostic nomogram for HCC patients with BM

**Fig. 5:**
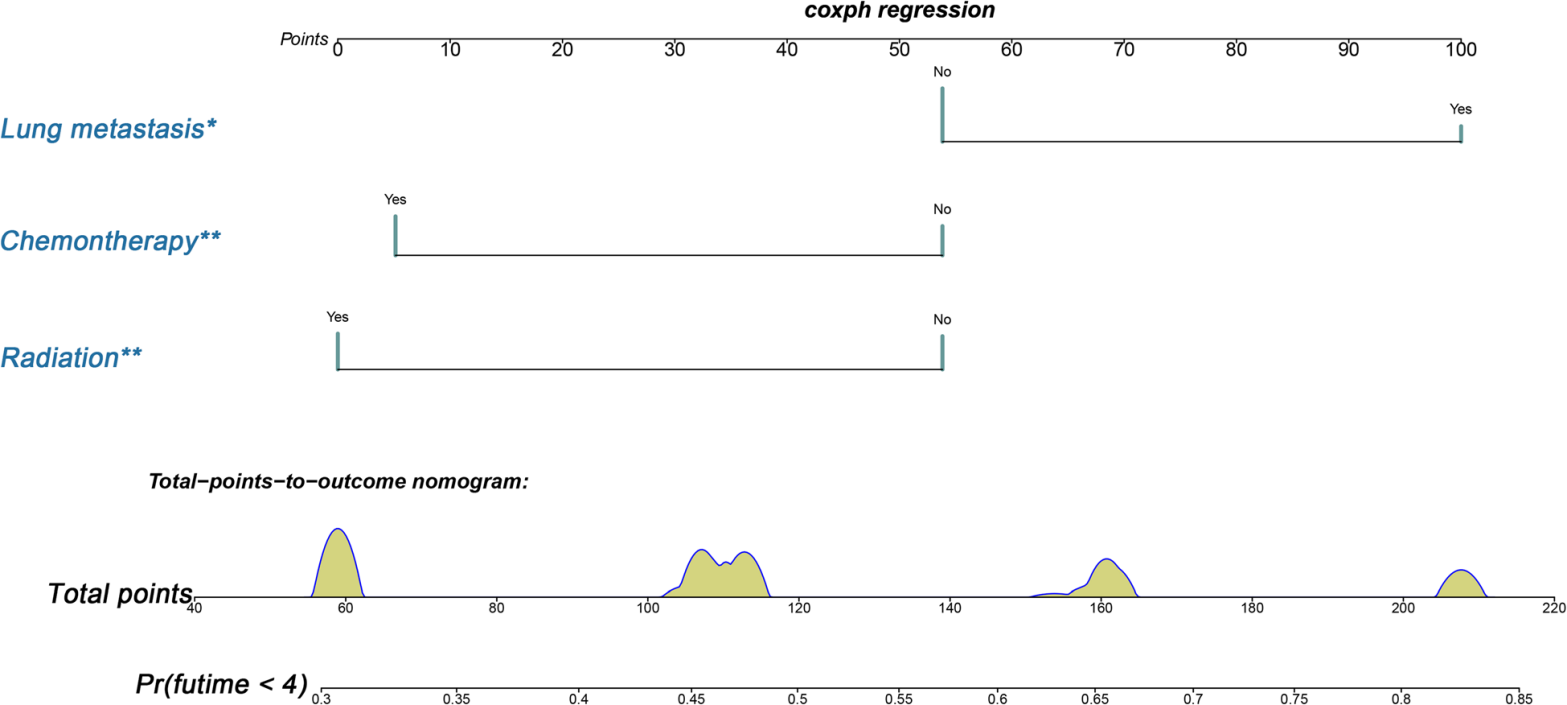
A prognostic nomogram for HCC patients with BM

**Fig. 6:**
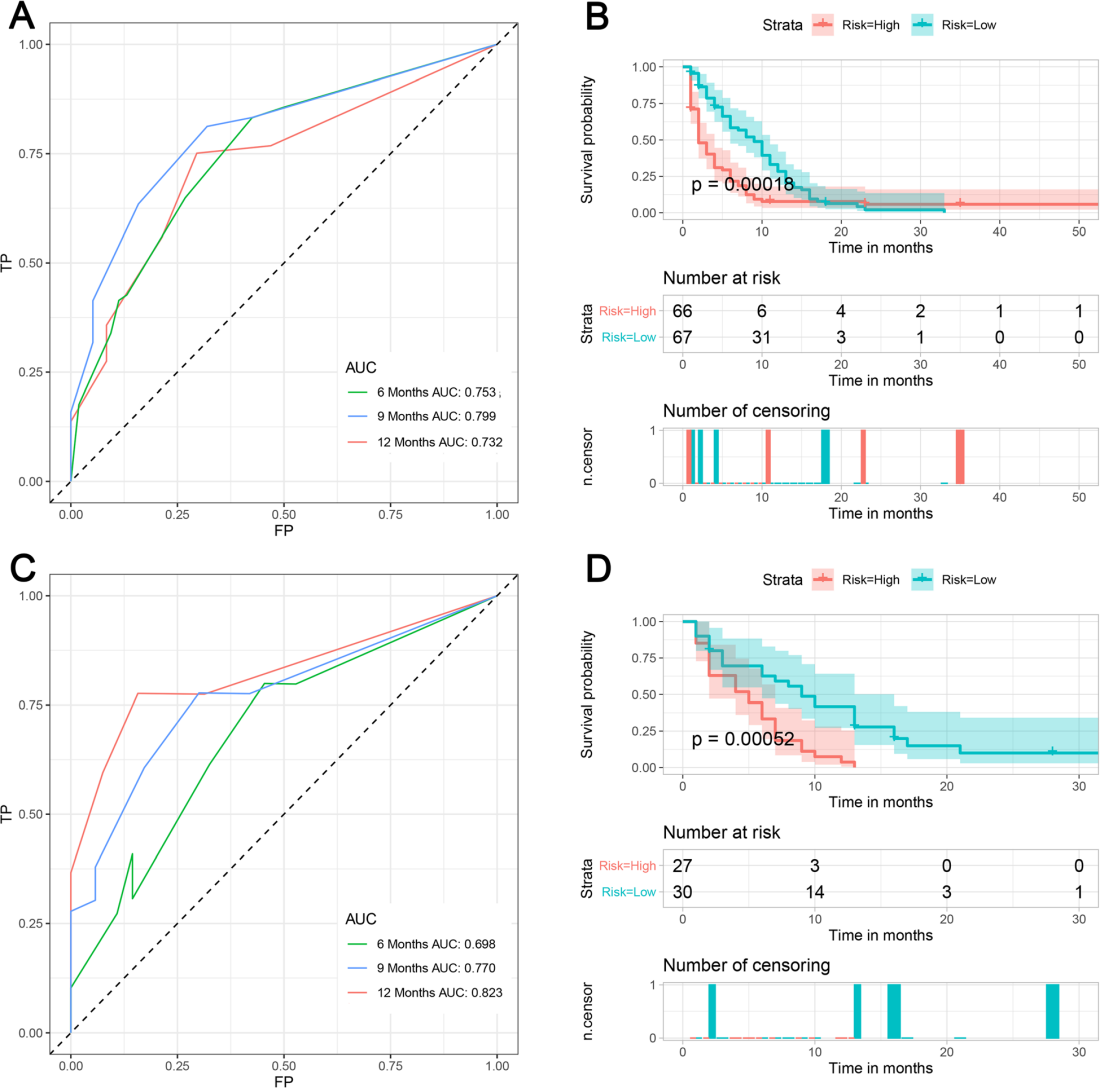
**a** Receiver operating characteristic curves of 6-, 9-, and 12-months in the training set; **b** The Kaplan-Meier survival curve of the training set; **c** Receiver operating characteristic curves of 6-, 9-, and 12-months in the testing set; **d** The Kaplan-Meier survival curve of the testing set

**Fig. 7:**
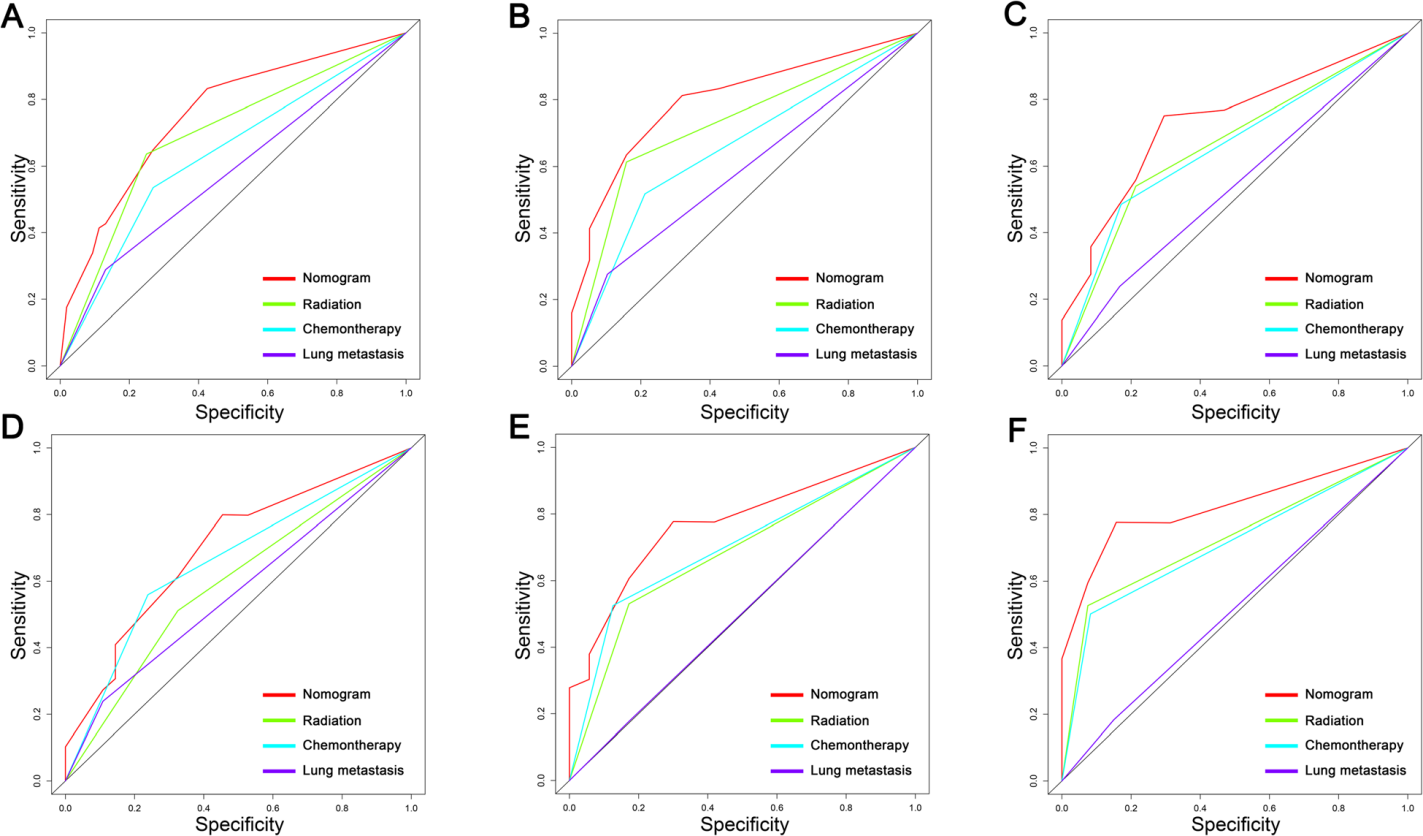
The receiver operating characteristic curves of nomogram and all independent predictors at 6- (**a**), 9- (**b**), and 12-months (**c**) in the training set and at 6- (**d**), 9- (**e**), and 12-months (**f**) in the testing set

**Fig. 8:**
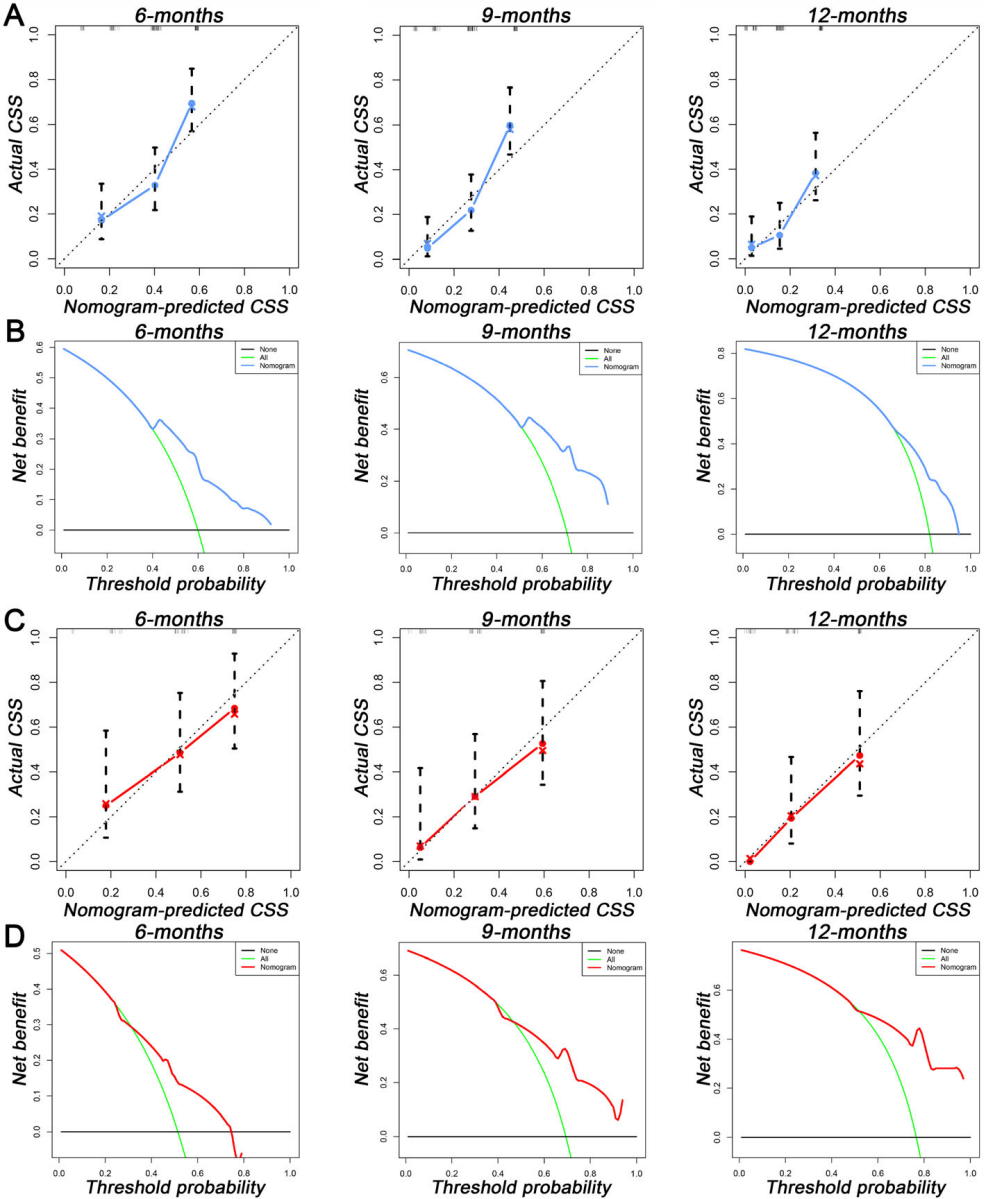
**a** The calibration curves of the nomogram in the training set; **b** the decision curve analysis of the nomogram in the training set; **c** The calibration curves of the nomogram in the testing set; **d** The decision curve analysis of the nomogram in the testing set

A prognostic nomogram was established based on three independent prognostic factors (Fig. 5). The AUCs of 6-, 9-, and 12-months were 0.753, 0.799, and 0.732, respectively (Fig. 6a). Afterward, we further compared the discrimination between nomogram and independent prognostic factors, and the results indicated the AUC of nomogram was higher than AUCs of all independent factors in 6-, 9-, and 12-months (Fig. 7a). In the testing set, the AUCs of 6-, 9-, and 12-months were 0.698, 0.770, and 0.823, respectively (Fig. 6c). Moreover, in the testing set, we can find that the discrimination of nomogram was also better than all independent prognostic factors in 6-, 9-, and 12-months. In addition, the Kaplan-Meier survival curve suggested that patients in the high-risk group have a worse prognosis than patients in the low-risk group (Fig. 6b and d). The calibration curves for the probability of 6-, 9-, and 12-month CSS also indicated a good consistency between nomogram-predicted CSS and the actual outcome (Fig.8a and c). In addition, the DCA curves showed that the nomogram had a good predictive efficiency for CSS of HCC patients with BM (Fig. 8b and d).

## Discussion

HCC is an aggressive tumor and prone to extrahepatic metastasis, occurring in 14.0–36.7% of patients [20]. Meanwhile, the detection of extrahepatic metastasis from HCC increased because of the development of survival and diagnostic modalities [21]. Bone is a common site of extrahepatic metastasis, and the incidence ranges from 2 to 25% in HCC patients [12]. In our study, we established a diagnostic nomogram for predicting the BM in newly diagnosed HCC patients and a prognostic nomogram for HCC patients with BM. By obtaining the data of several easily accessible variables on the nomogram of each HCC patient, the total score can be calculated. Then, the risk of BM can be easily identified on the nomogram, which can provide guidance for further clinical management. Similarly, the prognosis of HCC patients with BM can be identified by prognostic nomogram. In our research, both nomograms demonstrated excellent performance in the risk assessment of BM and the survival prediction of HCC patients with BM, which will make the individualized clinical decision and surveillance more accurate.

Although the prognosis is extremely poor in HCC patients with BM, the early detection of BM could be crucial for HCC patients to receive appropriate therapy [22]. Therefore, it appears to be important for clinical decision-making to explore the risk factors for BM from HCC patients. In the molecular level, the expression of Chemokine receptor CXCR4 [23], MicroRNA-34a, [24] and LncRNA34a [25] were identified to be associated with BM in HCC patients. Nevertheless, these biomarkers were difficult and unpractical to apply immediately to clinical decisions. In addition, as for some practical clinical features, it was reported that marital status, T stage, N stage were risk factors for BM from HCC [12]. However, to date, no predictive model has been established, which means that the individual risk of BM cannot be identified by combining all independent BM-related predictors. In our study, the results showed that sex, grade, T stage, and N stage were the significant predictors for BM from HCC. The association between these factors and BM in HCC patients has been reported in previous researches. The association between tumor differentiation and TNM stage and BM in HCC patients has been confirmed in the previous study [26]. Another improvement of the nomogram was that the discrimination of nomogram was confirmed higher than any single predictors, which also showed the importance of a comprehensive predictive model.

In addition, our research showed that HCC patients with BM with lung metastasis, absence of chemotherapy, and absence of radiotherapy had unfavorable prognosis. Based on three independent prognostic factors, a nomogram was established. The results indicated that the nomogram can serve as an effective tool to identify high-risk patients. Similarly, the relationship between lung metastasis, chemotherapy, and radiotherapy and the prognosis in HCC patients have been widely reported in previous researches. In 2017, Yang et al. reported that multiple tumors and extrahepatic invasion were the independent adverse prognostic factors for HCC patients [27]. Therefore, as a common extrahepatic invasion site, patients with pulmonary metastasis were worse prognosis than patients without it. HCC with extrahepatic spread is considered to be in the advanced stage, and the therapeutic recommendation for this stage is oral sorafenib treatment [3]. Sorafenib had a positive effect on the survival of patients with advanced HCC [28]. Meanwhile, systemic chemotherapy with doxorubicin, gemcitabine or combined regimens for palliative care also improved HCC patients survival [3]. It was consistent with those reports that the absence of chemotherapy could lead to poor prognosis for HCC patients with BM and be an independent prognostic factor in our study. Generally, radiotherapy is a kind of treatment for uncomplicated symptomatic bone metastasis from HCC, aimed at palliation of symptoms [22, 29]. In the previous reports, radiotherapy was shown to provide effective palliation for patients with painful BM from HCC [9, 30]. In our research, radiotherapy showed favorable CSS in multivariate Cox regression analysis. Therefore, we recommend paying attention to the possibility of lung metastasis in patients with BM of HCC. For the sake of good prognosis, clinical treatment in HCC patients with BM could tend to be radiotherapy and chemotherapy. And further studies of significant prognostic factors for CSS in HCC with BM are necessary.

However, several limitations to our study should be noted. First, limited patients (*N* = 190) may result the possible error. Second, the information collected in the SEER database was about the disease at the first diagnosis, which meant that the bone metastasis occur in the latter stage cannot be recorded. Third, this was a retrospective study in which selection bias existed inevitably, and the information about detailed treatment was not available in the SEER database.

## Conclusions

Our study showed that sex, grade, T stage, and N stage were the risk factors of BM from HCC. As for HCC patients with BM, lung metastasis, chemotherapy, and radiotherapy were independent prognostic factors for CSS. Two nomograms we created may be individual, convenient, and more intuitive visual tools for risk assessment and prognostic prediction for BM from HCC.

## Data Availability

The dataset from SEER database generated and/or analyzed during the current study are available in the SEER dataset repository (https://seer.cancer.gov/).

## Abbreviations

BM: Bone metastasis
HCC: Hepatocellular carcinoma
SEER: Surveillance, Epidemiology, and End Results
ROC: Receiver operating characteristic
DCA: Decision curve analysis
CSS: Cancer-specific survival
AUC: Area under the curve
